# Sugar Intake, Obesity, and Diabetes in India

**DOI:** 10.3390/nu6125955

**Published:** 2014-12-22

**Authors:** Seema Gulati, Anoop Misra

**Affiliations:** 1 Diabetes Foundation (India), Safdarjung Development Area, New Delhi 110016, India; E-Mail: seemagulati2007@gmail.com; 2 Center of Nutrition & Metabolic Research (C-NET), National Diabetes, Obesity and Cholesterol Foundation (N-DOC), Safdarjung Development Area, New Delhi 110016, India; 3 Fortis C-DOC Center for Excellence for Diabetes, Metabolic Disease and Endocrinology, New Delhi-110048, India; 4 Fortis Hospital, Vasant Kunj, New Delhi 110067, India

**Keywords:** type 2 diabetes mellitus, obesity, sugar, India

## Abstract

Sugar and sweet consumption have been popular and intrinsic to Indian culture, traditions, and religion from ancient times. In this article, we review the data showing increasing sugar consumption in India, including traditional sources (jaggery and *khandsari*) and from sugar-sweetened beverages (SSBs). Along with decreasing physical activity, this increasing trend of per capita sugar consumption assumes significance in view of the high tendency for Indians to develop insulin resistance, abdominal adiposity, and hepatic steatosis, and the increasing “epidemic” of type 2 diabetes (T2DM) and cardiovascular diseases. Importantly, there are preliminary data to show that incidence of obesity and T2DM could be decreased by increasing taxation on SSBs. Other prevention strategies, encompassing multiple stakeholders (government, industry, and consumers), should target on decreasing sugar consumption in the Indian population. In this context, dietary guidelines for Indians show that sugar consumption should be less than 10% of total daily energy intake, but it is suggested that this limit be decreased.

## 1. Introduction

The most popular sweetener in the world, sugar, was invented in India. There is reference to sugarcane cultivation and the preparation of sugar in an Indian religious text, the *Atharva Veda*. The word sugar is a derivative of “*sarkara*”, meaning gravel in Sanskrit. Sugar became known to the world when the army of Alexander the Great came to India in 327 BC. Interestingly, they were surprised to see another alternative to honey to sweeten food, and described it as a “reed that gives honey without bees” [1].

Traditionally, any occasion in India is celebrated with intake of sweets. Also, it is customary to “sweeten the mouth” after every meal, any joyous occasion, religious festival, social gathering, *etc*. It is considered mandatory to offer sweets to the gods on every religious occasion (e.g., it is believed Lord Ganesha, who is worshiped first in all religious occasions, is fond of *ladoos* (made by frying a batter of gram flour and *ghee* in small pearl-size drops and then mixing with sugar syrup, this mixture is given a round shape). Indian religious offerings mostly contain five *amrits* (elixirs) like milk, curd, *ghee* (clarified butter), honey, and sugar; these indicate the importance of sugar not only as a food item but also as intrinsic to the Indian way of life. While sugar is of considerable cultural and hedonic relevance in India, nutritionally it provides only “empty” calories (1 g of sugar gives 4 kcal). It lacks the natural minerals which are present in the beet root or sugarcane.

**Figure 1:**
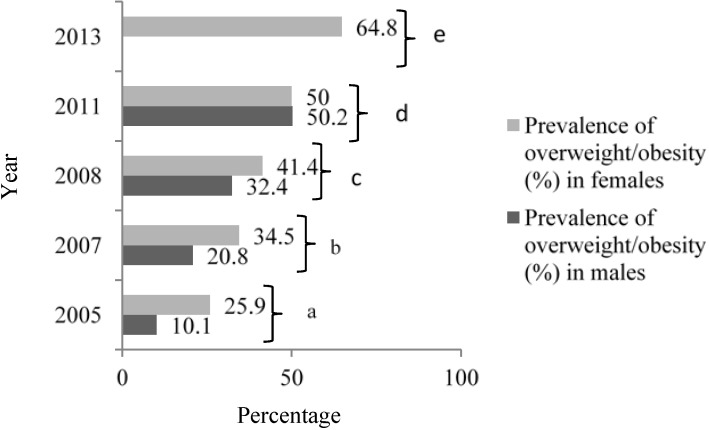
Prevalence of overweight/obesity in India, 2005–2013.

**Figure 2:**
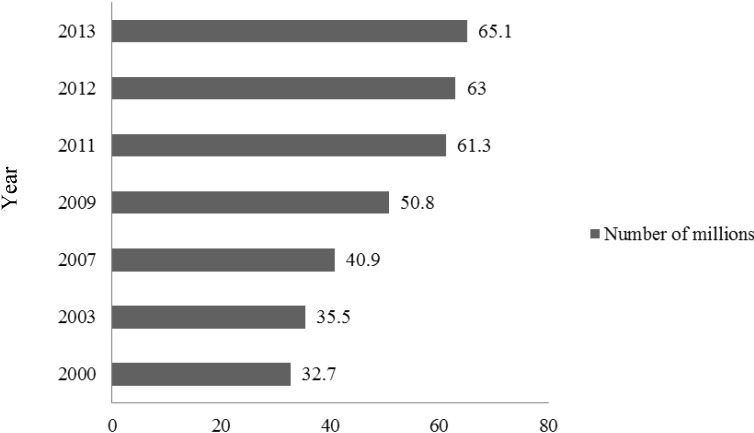
Number of patients with diabetes in India, 2000–2013.

Source: Data from International Diabetes Federation Atlas 2000–2013 [4,11,12,13,14,15,16].

There is a strong relationship between calorie intake and obesity. In India, the prevalence of obesity is increasing at a rapid pace (Figure 1) due to an increase in energy intake owing to increased purchasing power and availability of high fat, energy-dense foods, along with reduction in the energy expenditure consequent to urbanization and mechanization [2,3]. Parallel to the rise in overweight and obesity, the prevalence of metabolic syndrome and type 2 diabetes mellitus (T2DM) is also increasing in India, and has reached epidemic proportions. India has more than 65 million diabetics, second only to China worldwide (Figure 2) [4].

Source [5]: a, Misra *et al.*, 2005 [6]; b, Gupta *et al.*, 2007 [7]; c, Chow *et al.*, 2008 [8]; d, Bhardwaj *et al.*, 2011 [9]; e, Gulati *et al.*, [10].

In this article, we review the data regarding sugar consumption in India and review potential relationships of sugar intake with obesity and metabolic perturbations in humans.

## 2. Search Strategy and Limitations

To identify articles documenting the sugar consumption trends in India, we carried out a literature search using the terms “sugar, sugar intake, sugar consumption in India, fructose, sucrose, harmful effects of sugar intake, obesity and T2DM in Indians” in the medical search database PubMed (National Library of Medicine, Bethesda, MD, USA) from 1966 to June 2014. A manual search of the relevant quoted references was also carried out from the retrieved articles. Data have also been taken from nutritional surveys in India and worldwide, websites and published documents of the World Health Organization (WHO), the Food and Agricultural Organization (FAO), and websites of industries related to sugar production. We have frequently referred to data from the National Sample Survey organization (NSSO), which are household surveys conducted by the National Sample survey office, a focal agency of the government of India, to collect statistical data on demographic, agricultural, health, nutritional, and other aspects that are vital for developmental planning.

It is important to note that, despite an elaborate literature search regarding sugar intake and its relationship with obesity and T2DM, only limited data are available from India. In such scenarios, we have supplemented data from various sources such as websites of industries producing sugar and sugar-containing products, *etc*. It must be noted that data from some national research organizations that have been referred to here may be fraught with errors, since most rely on questionnaires based on subjective responses; this may result in under-reporting of dietary intake and incomplete information. Moreover, many of these data are based on inconsistent and differing methodologies of data collection.

### Definitions

The expert consultations organized by the WHO and the FAO of the United Nations, as well as the scientific updates undertaken by the WHO [17,18,19], have adopted a classification of sugars and clarified definitions of various groups of sugars including the category of “free sugars” (Table 1). This classification enables a more standardized approach to examining potential adverse health effects.

**Table 1:** Classification of dietary sugars.

Sugars: Subgroups	Principal Components
Monosaccharides	Glucose, fructose, galactose
Disaccharides	Sucrose (glucose and fructose), lactose (glucose and galactose), maltose (glucose and glucose), trehalose (glucose and glucose)
Free sugars	All monosaccharides and disaccharides added to foods by the manufacturer, cook, or consumer; sugars naturally present in honey, syrups, and fruit juices

Source: [19,20]. Please note that sugar alcohols have not been included here.

The term “added sugar” is sometimes used interchangeably with “free sugar” but is considered to include sugars and syrups added to foods during processing, food preparation, or at the table, but does not include honey or fruit juices [20].

Sugar-sweetened beverages (SSBs) include the full spectrum of aerated drinks, fruit drinks, and energy and vitamin water drinks containing added sugars. Many of these beverages are sweetened with high fructose corn syrup (HFCS), the most common added sweetener in processed foods and beverages, and some with sucrose or fruit juice concentrates. The HFCS that is commonly used in beverages contains 55% fructose and 45% glucose, while sucrose or table sugar consists of 50% fructose and 50% glucose.

In the Indian context, available databases do not define sugars clearly; however, from the data breakdown it appears that “sugar” means white sugar, honey, or brown sugar but not syrups and “traditional sugars” such as jaggery (also called *gur* in India) and *khandsari*. These “traditional sugars” are produced from sugarcane in addition to sugar. Jaggery is obtained by boiling clarified sugarcane juice until a solid residue is left after evaporation. It usually contains 65%–85% sucrose and is also source of calcium, potassium, and iron. *Khandsari* is a finely granulated crystallized sugar that contains 94%–98% sucrose. It is less refined than sugar and retains some calcium. The commonly consumed sources of sugars in India are listed in Table 2.

**Table 2:** Commonly consumed food articles in India containing natural or added sugars.

Main Meals	Snacks	Beverages	Additional
all carbohydrates: rice, wheat, buckwheat, oats, millets, barley, breads, *etc.*; sugar/jaggery stuffed Indian bread, yogurt, vegetables, *etc*.	Indian sweets (*halwa* ^a^, *kheer* ^b^, *etc.*), *kulfi* ^c^, *chikki* ^d^, puddings, fruit cakes, cookies, ice creams, processed foods, *etc*.	sugar cane juice, *Shikanjvi* ^e^, sweetened *lassi* (buttermilk), *sharbat* ^f^, *aam panna* ^g^, milkshakes, fruit juices, sugar-sweetened beverages (SSBs), *etc*.	sweet *chutneys* ^h^, pickles, *aamras* ^i^, *murabbas* ^j^, honey, *khandsari* with g*hee* ^k^, jams, tomato ketchup.

^a^ made by roasting semolina in a lot of *ghee* and then adding water, sugar, and nuts; ^b^ sweet dish made from boiling rice with milk, sugar, cardamom, saffron, and nuts; ^c^ frozen dairy desert like ice cream; ^d^ sweet candy generally made from peanuts and jaggery; ^e^ sweetened lime water; ^f^ sweet beverage prepared with fruit juice or flower petals; ^g^ beverage prepared from raw mango, mint leaves, sugar, and salt; ^h^ thick sauce of Indian origin that contains fruits/vegetables, sugar, and spices and is used as a condiment; ^i^ made by adding sugar to mango pulp; ^j^ candied fruits/vegetables; ^k^ clarified butter. SSBs, Sugar-sweetened beverages.

## 3. Sugar Consumption in India

Data from the India sugar trade industry (2013) show that India is the second largest (after Brazil) producer and largest consumer of sugar in the world [21]. India is also the largest producer of *khandsari* and *gur* [22]. It is important to mention here that as per the NSSO report, per capita consumption of jaggery and *khandsari* was 8.72 kg per annum in 2001 [23]. There is no recent information in government reports on consumption of these “traditional sugars.” Reports from the sugar industry indicate that almost 32% of sugarcane is still utilized in the production of jaggery and *khandsari* [22]. Also, per capita consumption of these “traditional sugars” dropped to approximately 5 kg per annum in 2011 [24].

**Figure 3:**
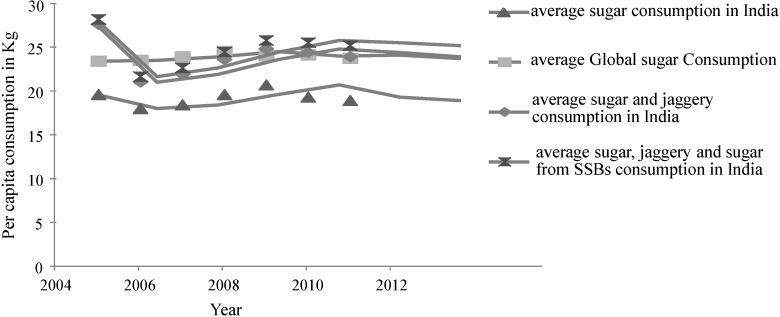
Trend line showing average intake of sugar globally and total sugar intake from various sources (“traditional sugars”: jaggery and *khandsari*; sugar and sugar from sugar-sweetened beverages) compiled for India.

Source: [24,25,26].

The following discussion and data (Figure 3, Table 3) have been based on the Sugar Year Book, reports from the sugar industry, and a recent article by Basu *et al.* [24,25,26].The per capita sugar intake is defined as the raw sugar consumption per person of a given country or territory. This is calculated based on the statistical disappearance of sugar in the country or territory after adjustment for trade and exports [27]. The assumption is made that the statistical disappearance of sugar is equal to consumption after adjusting for utilization for non-human consumption. Indian sugar production exceeded 27 million tons during 2012–2013, a jump from 15 million tons in 2005 (Table 3). Overall sugar intake has not changed from 2008 to 2011; however, a slight decrease in sugar intake from 19.6 kg in 2005 to 18.9 kg in 2011 has been recorded. Interestingly, while intake of “traditional sugars” has declined, an increase in the intake of sugar from SSBs has been recorded. It is interesting to note that when consumption from jaggery/*khandsari* and SSBs are added to that of white sugar [24,25,26,28], the “total” sugar intake in Indians exceeded the average global per capita consumption (Figure 3).

On the other hand, in comparison to data from sources indicated previously [26], data from the NSSO for 2009/2010 [29] included SSBs (Table 4) under “miscellaneous food, food products, and beverages” (“Misc. food, *etc.*”). These data show that in comparison to “sugar and honey,” the contribution from “Misc. food” to total calories consumed was more, both in rural and urban populations in the year 2009–2010 (Table 4).

**Table 3:** Sugar production, consumption, and stocks in India, 2005–2011.

Sugar	2005	2006	2007	2008	2009	2010	2011
Production (tons raw value) ^a^	15,215,826	22,346,546	29,090,294	25,936,000	15,654,974	21,150,846	27,960,000
Consumption Tons (raw value) ^a^	20,109,500	20,109,500	20,878,009	22,550,000	24,131,400	22,827,000	23,133,000
Stocks (tons raw value) ^a^	6,214,255	7,252,868	12,730,783	11,886,102	7,881755	8,112,199	10,101,903
Per capita sugar consumption (kg raw value) ^a^	19.6	18.0	18.4	19.6	20.7	19.3	18.9
Per capita sugar consumption (kg) from SSBs ^b^	0.48	0.55	0.64	0.73	0.84	0.96	1.11
Per capita jaggery and *khandsari* consumption (Kg) ^c^	8.0	3.0	3.5	4.0	4.1	5.1	5.0
Per capita consumption of sugar, jaggery, *khandsari*, and sugar from SSBs	28.08	21.55	22.54	24.33	25.64	25.36	25.01
Per capita global consumption average ^a^	23.4	23.5	23.9	24.5	24.0	24.1	23.7

Source: ^a^ [25]; ^b^ [26]; ^c^ [24]. SSBs, Sugar-sweetened beverages.

**Table 4:** Percentage breakdown of “sugar and honey” and miscellaneous foods: 2009–2010.

Population	% Share of Non-Cereals in Calorie Intake	% Share of Calorie Intake from Non-Cereals Contributed by Food Group							
		Roots and Tubers	Sugar and Honey	Misc. Foods *	Pulses, Nuts and Oil Seeds	Vegetables and Fruits	Meat, Eggs and Fish	Milk and Milk Products	Oils and Fats
Rural	39.6	9	11	20	11	7	3	16	23
Urban	49.6	6	10	19	12	8	3	17	25

Source: Government of India, National Sample Survey Organization, 2010 [29]; * Misc. foods: miscellaneous, processed foods, and beverages.

**Figure 4:**
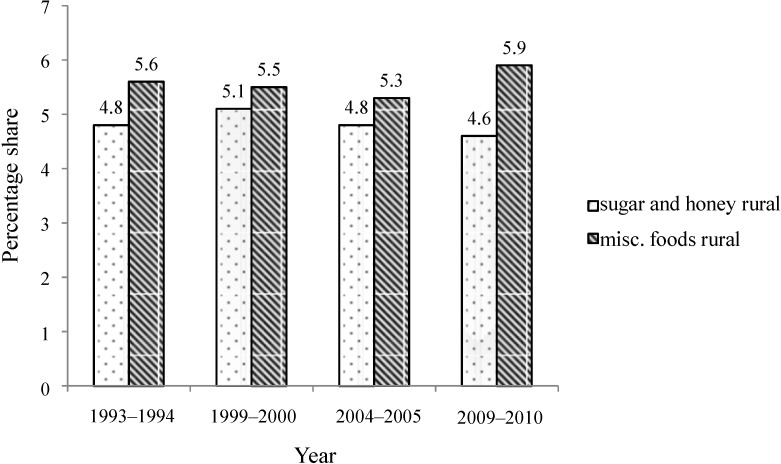
Percent share of total calories from “sugar and honey” and “misc. foods” in rural population of India, 1993–2010.

Source: Government of India, National Sample Survey Organization, 2010 [29]; Misc. foods: miscellaneous, processed foods, and beverages.

**Figure 5:**
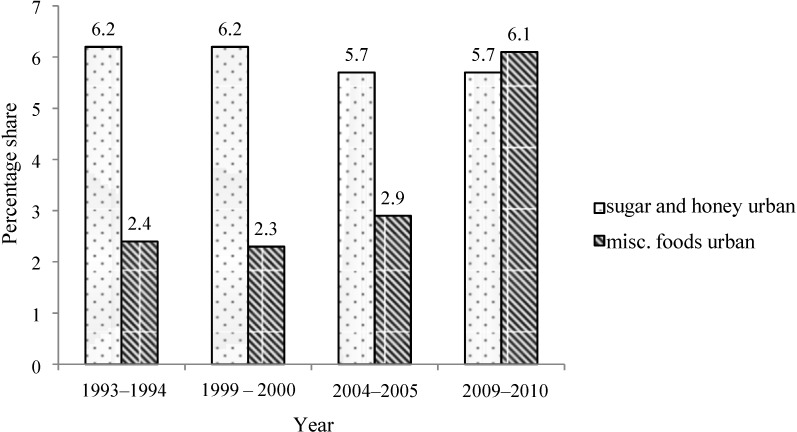
Percent share of total calories from “sugar and honey” and “Misc. foods” in urban population of India, 1993–2010.

Source: Government of India, National Sample Survey Organization, 2010 [29]; Misc. foods: miscellaneous, processed foods, and beverages.

Importantly, while a slight reduction in “sugar and honey” consumption has been recorded from 1993 to 2010 in rural and urban areas, there is a substantial increase in sugar-containing food items (“Misc. foods”) over time, especially in urban areas (Figure 4 and Figure 5).

### 3.1. Sugar-Sweetened Beverages

**Figure 6:**
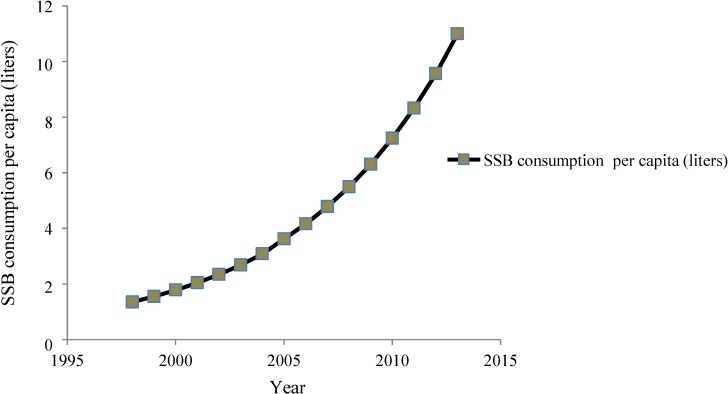
SSB consumption in liters per capita in India, 1998–2013.

Source: Date from [26]. SSB, Sugar-sweetened beverage.

**Figure 7:**
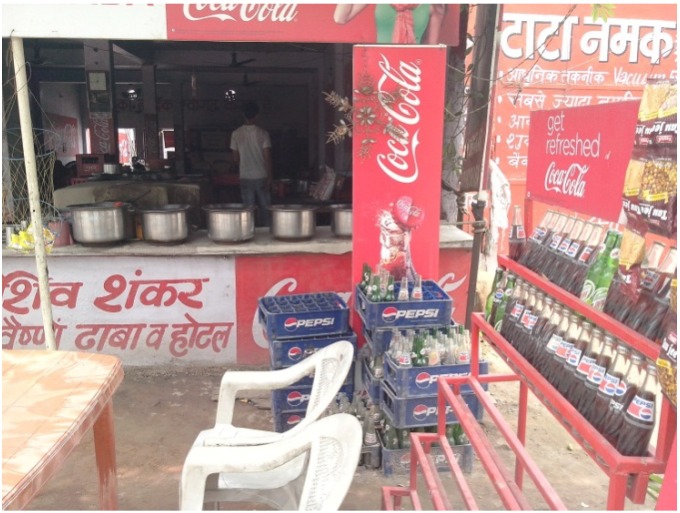
Bottles of sugar-sweetened beverages stacked outside a small shop (*dhaba*) on the national highway (NH-58), 60 km from Delhi.

SSBs constitute a significant contribution as the third largest industry in India after packed tea and biscuits, attracting direct foreign investment of over $1 billion in recent years. Production of SSBs was about 6.6 billion bottles in 2001–2002 in India [30]. SSB sales in India have increased by 13% per year since 1998, exceeding 11 liters per capita per year [28] (Figure 6). Easy availability of SSBs in rural and urban areas significantly contributes to higher per capita consumption. There is an increasing presence of *dhabas* (small shops) selling SSBs on all roadsides, particularly on highways (Figure 7). Furthermore, the beverages that people consume in India apart from SSBs like milkshakes, sweetened buttermilk, *etc.* are high in calories and glycemic load.

**Figure 8:**
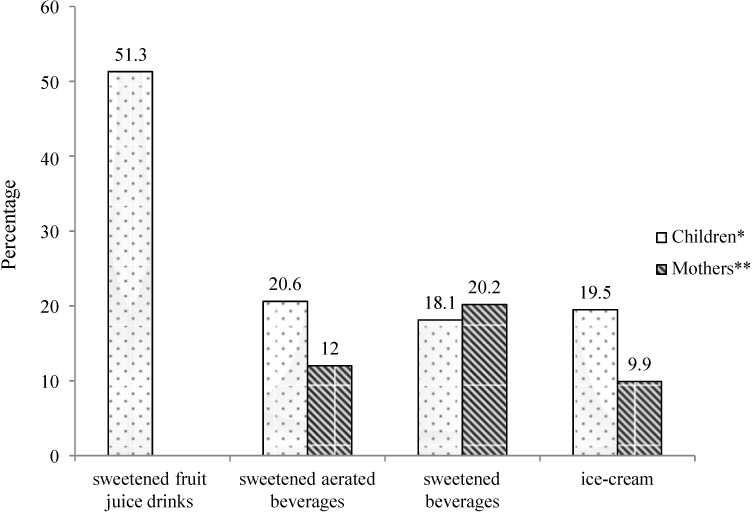
Percentage of children and mothers consuming different sweetened food items ≥4 times/week.

Source: * data from [10]; ** unpublished research data; data not available for consumption of sweetened fruit juice drinks for mothers.

The consumption pattern of sweets and beverages is rapidly changing among children as well. Importantly, SSBs and other sugar-containing high calorie foods are easily available within and around school premises. In a study conducted by our group of 1800 school children aged 9–18 years and their mothers, from four cities in India—Delhi, Bangalore, Pune, and Agra (methodology explained elsewhere) [10]—in 2013, we showed a high consumption pattern of sweetened food items among children and their mothers (Figure 8). The study highlighted the role of mothers in deciding the food choices of children and reported a strong association between the dietary intake of children and their mothers (*p* ≤ 0.001) for all the studied sweetened food items [10]. The study also showed that any food or food preparation was considered “healthy” if it was “hygienically” prepared. Thus, mothers preferred packaged foods, including bottled beverages, over restaurant food. Furthermore, the results showed that the consumption of food among children is influenced by television advertisements, peer pressure, and the “fashion” for consuming westernized foods [10]. Another study by our group [31] showed consumption pattern of colas among children and recorded consumption of approximately 1.8 cans of cola per week (540 mL/week; 1 can or 300 mL = 132 kcal and 33–40 g sugar) among children and adolescents in urban India, which could result in nearly 1.3 kg (3 lb) of weight gain per child per year.

In an important recent article by Basu *et al.* [26], variation in SSB consumption levels was studied utilizing data from the Indian Migration Study (IMS) (*n* = 7049 men and women). Among the 390 kcal/person/day typically consumed from beverages among surveyed Indians, approximately 12% (46 kcal/person/day) were contributed by SSBs. Consumption varied from 9% (37 kcal) among the older cohort of 45–65-year-olds to 13% (52 kcal) of overall beverage consumption among the younger 25–44-year-old cohort, and was roughly equal among urban (12%, 48 kcal) and rural populations (12%, 45 kcal). SSBs composed 14% of beverage calories among the poorest income tertile and 12% among the wealthiest tertile. However, overall beverage calories were lowest among the poor (310 kcal/person/day) *versus* the wealthiest tertile (404 kcal/person/day), hence the absolute consumption varied insignificantly by wealth (44 *versus* 47 kcal/person/day).

### 3.2. Indian Sweets

**Figure 9:**
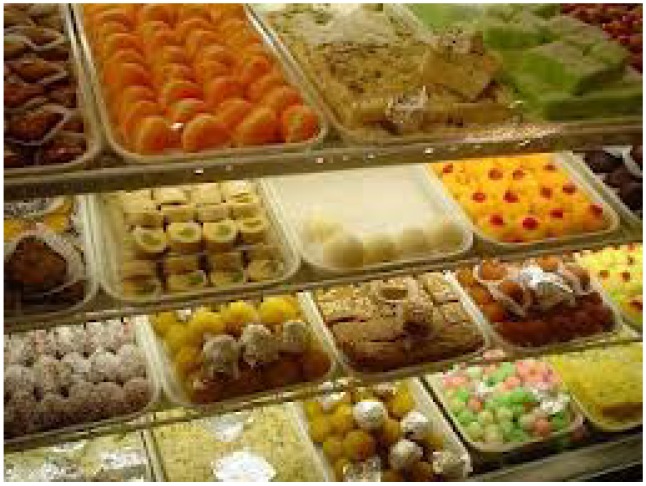
Indian sweets displayed in a commercial outlet in Delhi (North India).

**Figure 10:**
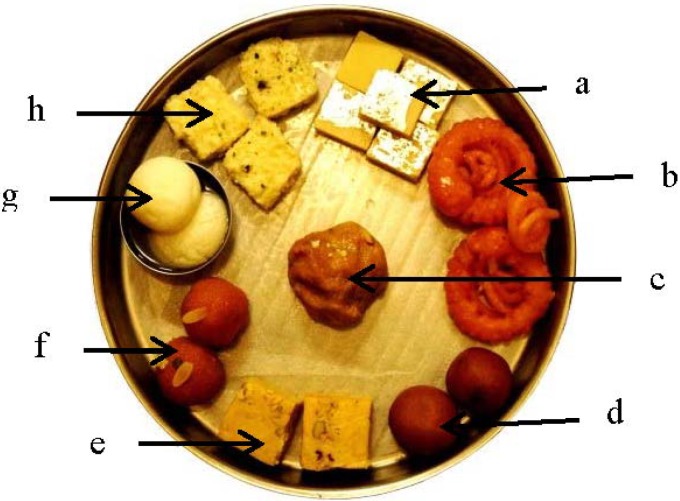
Photograph of commonly consumed North Indian sweets.

^a^ *Barfi* is made with condensed milk and sugar, cooked until it solidifies; ^b^ *Imarati/Jalebi* is made by deep-frying a refined wheat flour batter in pretzel or circular shapes, which are then soaked in sugar syrup; ^c^ *Sooji halwa* is made by roasting semolina in a lot of *ghee* and then adding water, sugar, and nuts; ^d^ *Gulab jamun* is prepared from milk solids kneaded into dough, sometimes with refined flour, and then shaped into small balls and deep fried. These are then soaked in sugar syrup; ^e^ *Soan Papdi* is prepared with gram flour, sugar, *ghee* (clarified butter), milk, and cardamom. It is usually square in shape, and has a crisp and flaky texture; ^f^ *Motichoor Ladoo* is made by frying a batter of gram flour and *ghee* in small pearl-size drops and then mixing with sugar syrup. This mixture is given a round shape; ^g^ *Rasgulla* are ball-shaped dumplings made out of dough kneaded from Indian cottage cheese and semolina, and then cooked in a syrup made of sugar; ^h^ *Kalakand* is made out of solidified, sweetened milk and cottage cheese.

In India, consumption of Indian sweets (Figure 9), which consist of a high concentration of sugar and fat, is high. Table 5 and Figure 10 clearly show that not only is sugar understandably high in Indian sweets but these are also high in saturated and *trans* fats (prepared using partially hydrogenated vegetable oils). The popular Indian commercial outlet selling Indian sweets, Haldiram’s, started in 1932 as one small shop in Rajasthan (west India) and has now expanded not only to almost all states of the country but also in 60 other countries.

Consumption of Indian sweets varies from region to region depending on climate, agriculture, and tradition. For example, sweets commonly consumed in north India are different from those consumed in south India. Other local practices also increase sugar consumption in meals, for example in the state of Gujarat (midwest India), there is a practice of adding sugar to all gravies, breads, curd, *aamras* (mango pulp), *etc*.

**Table 5:** Calorific value of some of the commonly consumed North Indian sweets.

Sweet(100 g)	Piece	Energy (kcal)	Fat (g)	Carbohydrate (g)	Protein (g)
*Barfi* ^a^	5	285	12	39	7
*Imarati/Jalebi* ^b^	2	500	18	80	4
*Sooji Halwa* ^c^	1/2 bowl	216	12.9	24.3	1.9
*Gulab jamun* ^d^	2	313	4.8	54.6	3.036
*Soan Papdi* ^e^	2	504	23	67	7
*Motichoor Ladoo* ^f^	2	362	26.6	29.6	3.3
*Rasgulla* ^g^	2	186	1.85	38	4
*Kalakand* ^h^	3	389	22.9	33.96	13.4

^a^ *Barfi* is made with condensed milk and sugar, cooked until it solidifies; ^b^ *Imarati/Jalebi* is made by deep-frying a refined wheat flour batter in pretzel or circular shapes, which are then soaked in sugar syrup; ^c^ *Sooji halwa* is made by roasting semolina in a lot of *ghee* and then adding water, sugar, and nuts; ^d^ *Gulab jamun* is prepared from milk solids kneaded into dough, sometimes with refined flour, and then shaped into small balls and deep fried. These are then soaked in sugar syrup; ^e^ *Soan Papdi* is prepared with gram flour, sugar, *ghee* (clarified butter), milk, and cardamom. It is usually square in shape, and has a crisp and flaky texture; ^f^ *Motichoor Ladoo* is made by frying a batter of gram flour and *ghee* in small pearl-size drops and then mixing with sugar syrup. This mixture is given a round shape; ^g^ *Rasgulla* are ball-shaped dumplings made out of dough kneaded from Indian cottage cheese and semolina, and then cooked in a syrup made of sugar; ^h^ *Kalakand* is made out of solidified, sweetened milk and cottage cheese.

## 4. Harmful Effects of Sugar Intake and Obesity/T2DM in Indians

Systematic reviews and meta-analyses of 20 controlled feeding trials in 344 participants by Sievenpiper [32] suggest that fructose more than glucose may increase total cholesterol, uric acid, and postprandial triglycerides under calorie matched conditions, especially at high doses. However, its effects on the atherogenic aspects of the lipid profile (low density lipoprotein cholesterol, apoB, non-high density lipoprotein cholesterol, total cholesterol: high density lipoprotein cholesterol ratio), insulin, and markers of non-alcoholic fatty liver appear to be no worse than those of glucose. Fructose may also have important advantages for body weight, glycemic control, and blood pressure over glucose. But overall, multiple short-term studies find that sugar intake leads to the following adverse events, mostly through accumulation of body fat [33] and intra-abdominal fat [34]: hyperuricemia [35], hypertriglyceridemia [36], insulin resistance [37], metabolic syndrome [37], diabetes [38], fatty liver [39], and high levels of free fatty acids [40].

High doses of fructose (>50 g/day at least) in humans have been implicated in insulin resistance, postprandial hypertriglyceridemia, intra-abdominal fat accumulation, and elevated blood pressure [34,37,41,42], mediated by high levels of non-esterified fatty acid (NEFAs) [43]. Increased portal delivery of NEFAs increase hepatic glucose production [44,45], impair β cell function [46], and cause hepatic steatosis. Interestingly, SSBs increase the risk of metabolic syndrome and T2DM not only through increasing adiposity but also by increasing the dietary glycemic load, which causes insulin resistance, β-cell dysfunction, and inflammation [47]. Specifically, risk of T2DM associated with SSB consumption in humans has been found to be statistically significant after adjustment for total energy consumption and body mass index (BMI) [48,49].

The above discussion suggests that sugar intake contributes to multiple metabolic disorders due to accrual of body fat, as well as directly through excess NEFAs, which in turn impair critical functioning of the liver, pancreas, and cellular functions.

**Figure 11:**
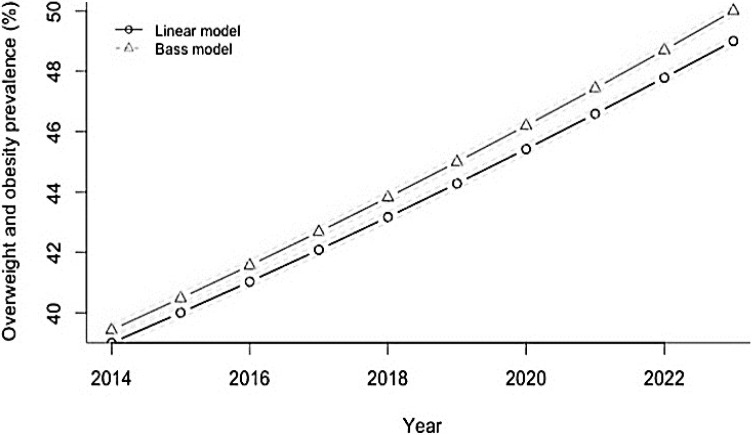
Projected trajectory of overweight and obesity in India, 2013–2023, if SSB consumption continues at the same rate.

Source: Adapted from [26] with permission. SSB, Sugar-sweetened beverage.

**Figure 12:**
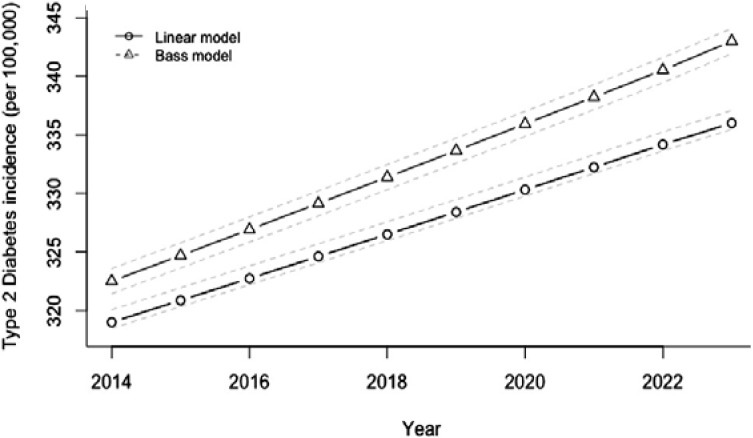
Projected trajectory of type 2 diabetes mellitus incidence in India, 2013–2023, if SSB consumption continues at the same rate.

Source: Adapted from [26] with permission. SSB, Sugar-sweetened beverage.

In this context it is important to mention here that Indians already have higher NEFAs, insulin resistance, hepatic steatosis, and dysglycemia than white Caucasians [50]. All these metabolic dysfunctions could be further exacerbated by indirect (through obesity) and direct effects on multiple metabolic organs. Importantly, Indians are increasingly consuming traditional Indian sweets along with SSBs, and westernized sugar-loaded food items, which are now easily available due to globalization. Although research data are lacking, it would not be irrational to presume that increasing intake of sugar/sugar containing products (Figure 11 and Figures 12) may parallel the rapid rise of obesity and T2DM in Indians. In this respect, it is important to note that Weeratunga *et al.* [27] analyzed data from 165 countries to study the associations between the prevalence of diabetes mellitus and per capita sugar consumption, utilizing data from International Diabetes Federation and from the Sugar Year Book. They showed a stronger association between diabetes prevalence rates and per capita sugar consumption in Asia (*p* < 0.001; Beta = 0.707) and South America (*p* = 0.010; Beta = 0.550) R2 = 0.568 as compared to the rest of the world. A strong positive correlation coefficient (0.599; *p* < 0.001) was observed between the prevalence of T2DM and per capita sugar consumption using data from all 165 countries. Asia had the highest correlation coefficient with a PCC of 0.660 (*p* < 0.001) and lower correlations were observed for Africa (PCC = 0.381; *p* < 0.007). The Eastern European region demonstrated a positive correlation between per capita sugar consumption and T2DM prevalence (PCC = 0.608; *p* < 0.036), whereas other European regions did not (Eastern Europe: PCC = 0.608, *p* < 0.036; Northern Europe: PCC = −0.165, *p* < 0.649, Southern Europe: PCC = 0.384, *p* < 0.218; Western Europe: PCC = 0.097; *p* < 0.836).

In this context, it is important to note that according to projections by Basu *et al.* [26], if SSB consumption is continued at the same rate then the Indian overweight and obesity prevalence (percentage of adults 24–65 with BMI > 25 kg/m^2^) would be expected to increase from 39% to 49% and the incidence of T2DM would be expected to rise in parallel from 319 to 336 per 100,000 per year over the period 2014–2023 (Figure 11 and Figure 12).

## 5. Potential Reduction of Diabetes and Obesity in India by Limiting Intake of SSBs

It is clear that a rapid increase in obesity and T2DM is expected in the next decade if SSB consumption is not curbed. In this context, Basu *et al.* [26] have suggested that a 20% soda tax may lead to a reduction of 3% in obesity (or prevent 11.2 million new cases), and a 1.6% decline in T2DM, or prevent 400,000 cases, over the decade 2014–2023. Incidentally, a recent budget for the year 2014–2015 in India has declared an increase in tax on SSBs by 5% [51].

## 6. Recommended Dietary Guidelines for Sugar Intake

A number of authoritative scientific associations including the American Diabetes Association [52], the WHO [53], and the Institute of Medicine of the National Academies [54] recommend reductions in consumption of SSBs and sugar for prevention of obesity and T2DM (Table 6).

**Table 6:** Statements from scientific associations supporting a reduction of sugar-sweetened beverages and sugar.

Association	Statement
American Diabetes Association	Consumption of sucrose should be minimized to avoid displacing nutrient-dense food choices. Avoid sugary drinks like regular soda, fruit punch, fruit drinks, energy drinks, sweet tea, and other sugary drinks. These will raise blood glucose and can provide several hundred calories in just one serving.
World Health Organization	Added sugar should be limited to <10% of a person’s caloric intake. Limit the intake of free sugars.
Institute of Medicine of the National Academies	Increase access to free, safe drinking water in public places to encourage water consumption instead of sugar-sweetened beverages.

Source: [52,53,54].

Recently, in view of the rising prevalence of obesity and related disorders globally, the World Health Organization’s expert panel recently (May 2014) recommended decreasing sugar intake to 5% of total calorie intake to combat obesity [55]. These guidelines are formulated based on analyses of all published scientific studies on the consumption of sugars and how these data relate to excess weight gain and tooth decay in adults and children. In 2002, the WHO recommended that sugar should be less than 10 percent of daily calories. The consensus dietary guidelines for Indians in 2011 recommend less than 10% of total calories from free sugars per day [56] (Table 7). In view of the increasing sugar intake and adverse metabolic phenotype of Indians, it would be prudent to decrease this limit.

**Table 7:** Recommendations on sugar intake for Indians (2011).

1. Free sugars should be less than 10% of total calories/day, which includes all added sugars and sugars present in honey, syrups, and fruit juices.
2. Alternatives to sweetened beverages can be water, skimmed buttermilk, tender coconut water, and low fat milk.
3. Indian sweets, puddings, ice creams, sweetened biscuits, cakes, pastries, and baked goods are high in added sugars and should be restricted.
4. Encourage reading of food labels to determine sugar content. Some of the names in the ingredients list for the presence of added sugars include brown sugar, corn syrup, dextrose, honey, malt syrup, sugar, molasses, and sucrose.

## 7. Proposed Prevention Strategies for Reduction of Sugar Intake

To achieve a reduction in free sugar intake in India, concerted efforts are needed from all stakeholders including policy makers, government, consumers, food manufacturers, and other agencies involved in food production (food and SSBs). The most important among these is a firm political will that should specifically focus on curbing the rising prevalence of obesity, T2DM, and cardiovascular diseases in India. Some of the strategies to reduce sugar intake have been listed below. These proposed strategies will not only help in reducing sugar intake but will also have a meaningful impact in reducing the incidence of obesity and T2DM in India.

*Government:*
  (1) Strict guidelines regarding sugar intake should be formulated.
  (2) Spread awareness among consumers and the medical establishment regarding the ill effects of high sugar intake.
  (3) Increasing taxation could be one of the strategies to curb increasing consumption of SSBs.
  (4) Sale of SSBs should be banned in school premises. Healthy alternative drinks should be made available to children.
  (5) Warning labels such as “Drinking beverages with added sugar(s) contributes to obesity, diabetes, and tooth decay” could be mandatory for SSBs.
  (6) Increase access to free, safe drinking water in public places, schools, and offices to encourage water consumption instead of SSBs.
  (7) Restriction of advertisements for commercial foods on television (during prime time and children’s programs).
  (8) Encouragement of transnational food companies to manufacture healthy snacks and beverages.
  (9) Decrease taxes on and prices of fruits, vegetables, nuts, and other healthy foods.
*Consumers:*
  (1) Inculcate healthy eating habits in children from early childhood.
  (2) Instead of sugar and fat-loaded sweets, opt for fresh fruits for dessert. Raisins and dates can also be consumed to “sweeten the mouth” post meals.
  (3) Avoid gifting sweets; instead go for nuts and fresh fruits, *etc*. The beneficial effects of nuts have been established for Indians [57].
  (4) Cut down sugar in coffee and tea.
  (5) Read food labels carefully. Avoid intake of processed and packaged foods as much as possible.
  (6) Cut down on intake of sweetened SSBs. Instead opt for natural drinks such as clean plain water, water with certain herbs such as basil, mint, *etc.*, coconut water, buttermilk, lemon water, *etc*.

## 8. Industry/Food Outlets

(1) Nutritional labeling should include information on sugars present in the product, written in a trenchant manner so as to be intelligible by common consumers.
(2) Nutritional information of the foods served in restaurants should also be given in the menu card.

It must be mentioned that while these steps will help in curbing the harmful effects of sugar in the body, such preventive strategies are incomplete without enhancing physical activity. It is particularly important to note that the physical activity guidelines for Indians have suggested more rigorous and lengthier workouts as compared to exi**s**ting international guidelines. The patterns and recommendations for physical activity for Indians have been discussed in recent publications [58,59,60].

## 9. Conclusions

Data suggest an increase in sugar consumption in India, both from traditional sources and from SSBs. Along with decreasing physical activity, this increasing trend of sugar consumption assumes more metabolic significance in view of the high tendency of Indians to develop insulin resistance, abdominal adiposity and ectopic fat deposition, hyperglycemia, and atherosclerosis. Prevention strategies, encompassing multiple stakeholders, should target on decreasing sugar consumption and thus limiting its harmful metabolic perturbations through direct effects, and effects secondary to the accumulation of fat. These strategies must be combined with an active lifestyle and physical activity.
